# Simultaneous Quantification of Multiple Quality Traits Hidden in Living White/Pigmented Rice Seeds Both at 2D and 3D Level

**DOI:** 10.3390/plants15203123

**Published:** 2026-10-13

**Authors:** Zihan Wu, Min Sun, Qiwen Liang, Zhiwei Deng, Siyuan Wang, Shubo Zhou, Liying Ma, Langtao Xiao, Yi Su

**Affiliations:** 1 Hunan Provincial Key Laboratory of Phytohormones and Growth Development, Hunan Agricultural University, Changsha 410128, China; wuzihan1997@jxstnu.edu.cn (Z.W.); sunmin260513@163.com (M.S.); liangqw96@163.com (Q.L.); siyuanwang@163.com (S.W.); 2 Department of Agriculture and Forestry, Hainan Tropical Ocean University, Sanya 572022, China; dengzhiwei406@163.com (Z.D.); zhoushubo@hntou.edu.cn (S.Z.); liyingma@hntou.edu.cn (L.M.); 3 College of Life Sciences, Jiangxi Science and Technology Normal University, Nanchang 330013, China

**Keywords:** quality trait, simultaneous quantification, living rice seed, pigmented rice, 3D level, rice standard, microCT

## Abstract

Rice quality mainly includes milling, appearance, nutrition, eating, cooking and physicochemical qualities. Rice quality/grade assessment depends on multiple indicators (traits) testing based on the recommended methods in the executing rice standards. Regretfully, the quality/grade assessments for white and pigmented rice do not follow a unified standard. The utilization of a series of independent methods means complex operations, higher time requirements and cost because of the hidden, subtle, and irregular characteristics of quality traits. Destructive husking and polishing are usually the prerequisite steps before quality assessment, leading to a loss of seed activity. Additionally, these methods fail to provide spatial information of the quality traits within intact seeds. In this study, microCT was employed to capture the cross-section images of living rice seeds. We performed contrast and segmentation processes by using image segmentation software. Multiple quality traits were simultaneously determined using one group of cross-section images from one microCT scan both in living white and pigmented rice seeds. This research also expanded the scope of quality-related traits and displayed their 2D and (or) 3D information (e.g., linearity, area, surface area and volume). Our achievements can guide the establishment of a new unified standard for white and pigmented rice at the 3D level, and it also shows great potential for application in phenotyping and breeding.

## 1. Introduction

Rice (*Oryza sativa* L.) serves as a staple food for over half of the global population [1,2]. Rice quality/grade is the key factor influencing consumer choices and market circulation [3,4,5]. The demand for higher quality rice represents a major issue in rice production. The primary components of rice quality include milling, appearance, nutrition, eating, cooking and physicochemical qualities [4,6]. Milling, appearance and nutrition qualities largely determine market acceptance and value, thus attracting more attention from breeders, farmers, producers and consumers [7].

Representative indicators reflecting milling quality include the whole kernel rate, brown rice rate and milling degree [8,9]. Appearance quality primarily includes the shape, size, transparency, chalkiness, color and defect [10]. According to the market standard, high-quality rice generally possesses the characteristics of a well whole kernel, low chalkiness and good transparency. Undesired chalkiness, the opaque/white part, is a major determinant of rice grade and negatively affects milling and cooking qualities [11,12].

Along with the improving the living level, consumers’ demands for rice quality, especially nutritional quality, are gradually increasing. People expect to obtain more nutrients other than starch, such as proteins, nucleic acids, vitamins, anthocyanins, mineral elements and dietary fiber [13,14,15,16]. The bran layer (containing pericarp, seed coat and aleurone layer) and embryo are the major resource of these components; a thicker bran layer and larger embryo in rice indicates higher nutritional value [17]. Recent studies support this structure–nutrition link, showing that thickened aleurone increases multiple nutrients [17] and that seed coat/aleurone thickness is associated with identifiable loci and molecular markers [18,19]. Pigmented rice and anthocyanin-enriched endosperm further indicate that colored rice tissues contribute antioxidant phytochemicals [20,21,22]. In recent years, people are increasingly preferring to consume brown rice (from white rice) and pigmented rice. More and more cultivars of red/black/purple rice are being placed on consumers’ dining tables. Crop scientists in China propose changing dietary habits to make black rice a staple food in our daily life [23].

In order to evaluate the rice quality, some methods based on naked-eye observation, ruler measuring, imaging, physics and chemistry are employed to determine the quality-related traits/indicators [24,25]. Personal visual inspection and digital imaging coupled with supporting software were developed to analyze the rice appearance and milling indicators [26,27]. Near-infrared (NIR) spectroscopy has emerged as a powerful tool for the quantitative prediction of chemical constituents, such as protein, moisture, amylose, and lipid in whole grains or flour [28,29,30,31,32,33]. Hyperspectral imaging technology was also introduced for a non-destructive and high-throughput quantification of rice quality [34,35,36]. FTIR spectroscopy combined with machine learning algorithms (ML) could potentially predict the nutraceutical traits of pigmented rice [37,38,39]. X-ray based microcomputed tomography (micro-CT/μCT) is a non-destructive imaging technique that can be used to generate consecutive cross-section images of a sample with a micrometer to sub-micrometer scale resolution [40]. According to the cross-sections and reconstructed 3D objects, some size-, area- and volume-based biological traits can be visualized and quantified both in vitro and in vivo; X-ray microscopy enables multiscale high-resolution imaging [41]. Our group employed μCT to develop a 3D visualization and quantification method of rice chalkiness in vivo in a previous report [42].

Currently, the executing standards for pigmented rice and white rice constitute relatively independent systems. White rice standards contain national standards and international standards, and they guide the testing of quality traits in endosperm. Pigmented rice standards are usually industry standards, and they only involve the appearance/shape and pigment content. In executing rice standards, rice quality assessment includes destructive sample preparation, such as husking, milling and powdering, and also involves a series of methods relying on naked-eye observation, manual measurement, 2D-image recognition and chemical reaction. In short, current rice quality/grade assessments are complicated, time-consuming, costly and trait-deficient. The results do not reveal the spatial information of quality traits.

In this study, cross-section images (DCM format) of living white/pigmented rice seeds were captured by microCT. Contrast and segmentation processes were optimized. Multiple structural traits, relating to appearance, nutrition and milling quality, were in silico segmented and analyzed in a 3D image segmentation software. The linearity, area, surface area and volume were simultaneously obtained from one group of cross-section images. The thickness of the bran layer and the size of the embryo in rice were evaluated as the indirect indicators of nutritional components. The results can also reveal their cubic shape and spatial location. Our work expanded the scope of quality-related traits and simplified the operational process of rice quality assessment. It will support the construction of a new unified standard for quality assessment both in white and pigmented rice at the 3D level, and it provides a convenient approach for phenotyping and breeding.

## 2. Results

### 2.1. Workflow for Visualization and Determination of Quality Traits

**Figure 1:**
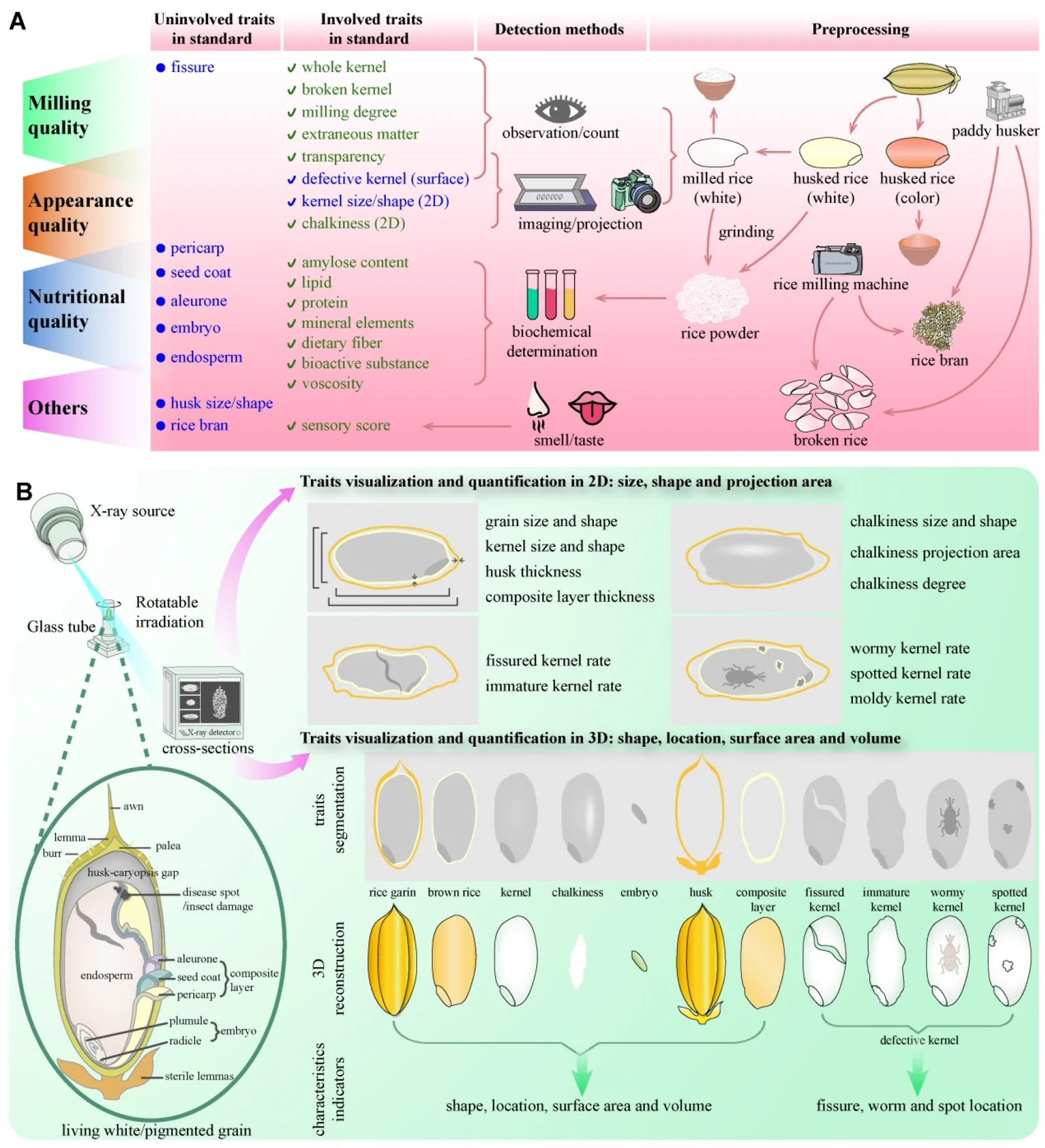
Workflow for visualization and determination of rice grain traits. (**A**) Current methods for detecting rice quality traits; (**B**) Workflow of μCT-based method for visualizing and quantifying rice quality traits.

A rice seed (paddy grain) consists of husk (including lemma, palea and sterile lemma) and caryopsis (i.e., brown rice or husked rice). Brown rice is composed of a bran layer (pericarp, seed coat and aleurone layer), embryo and endosperm. Milled rice (polished rice) generally refers to the endosperm, which is the major edible part and calorie source as a staple food. The quality assessment in white rice standards is essentially targeted at the endosperm. Rice bran was obtained by husking and milling rice, consisting of the husk, outer layer of caryopsis, germ/embryo, and broken kernel. Generally, the pericarp, seed coat and aleurone layer are difficult to separate structurally. These three parts are rich in lipids, protein, vitamins, mineral elements, dietary fiber, proanthocyanidins, anthocyanins and flavonoids both in pigmented and white rice. The embryo is another nutrient-rich organ containing nucleic acids, amino acids, lipids and other bioactive substances. According to the executing rice methods, only a portion of the rice quality traits can be determined. Different standards are being implemented and different indicators are considered for white rice and pigmented rice (Figure 1A, Table 1).

Recommended methodologies in rice standard for assessing rice grain quality traits (e.g., visual inspection, 2D imaging, or destructive sectioning) exhibit an obvious limitation and higher error in quantifying volume-based traits. These methods fail to achieve non-destructive quantification for detailed phenotypic characteristics particularly in the pericarp, seed coat, aleurone layer and embryo. Similarly, a comprehensive assessment of key pericarp traits in pigmented varieties (including thickness, volume, and surface area) remains technically challenging (Figure 1A, Table 1). The reliable non-invasive detection of internal fissure and defects caused by insect damage, pathogenic microorganism invasion and failed filling remains a persistent challenge for current approaches (Figure 1A).

To address these gaps, micro-CT was implemented in this study. Individual grains representing 30 white and 10 pigmented rice cultivars were subjected to volumetric imaging. This process achieved exceptional spatial resolution, enabling the capture of the complete structural details of each seed, including the husk, pericarp, endosperm and embryo (Figure 1B). Subsequent sophisticated image processing was essential to extract meaningful biological information from the complex volumetric datasets (Figure 1B). These coefficients directly correlate with tissue density variations. Phenotype analysis generates quantitative morphological and structural data from segmented 3D volumetric data (Figure 1B, Table 2).

This comprehensive analysis generates a series of 2D/3D metrics, such as dimensional parameters (length, width and thickness) and volumetric properties (surface area and volume) for the whole grain and kernel, a detailed quantification of chalkiness characteristics (surface area, volume, chalkiness degree and chalky rice rate), pericarp features (surface area, volume and thickness), and embryonic dimensions (size, shape and volume). Additionally, it facilitates the objective identification of internal fissure and other defects (Figure 1B, Table 2). This study establishes a robust, non-destructive methodology, enabling the simultaneous quantification of multiple quality traits hidden in living white/pigmented rice seeds both at the 2D and 3D level. It is expected to become a cornerstone methodology for food quality assessment and crop breeding.

### 2.2. Appearance Traits Can Be Detected at 2D and 3D Level

**Figure 2:**
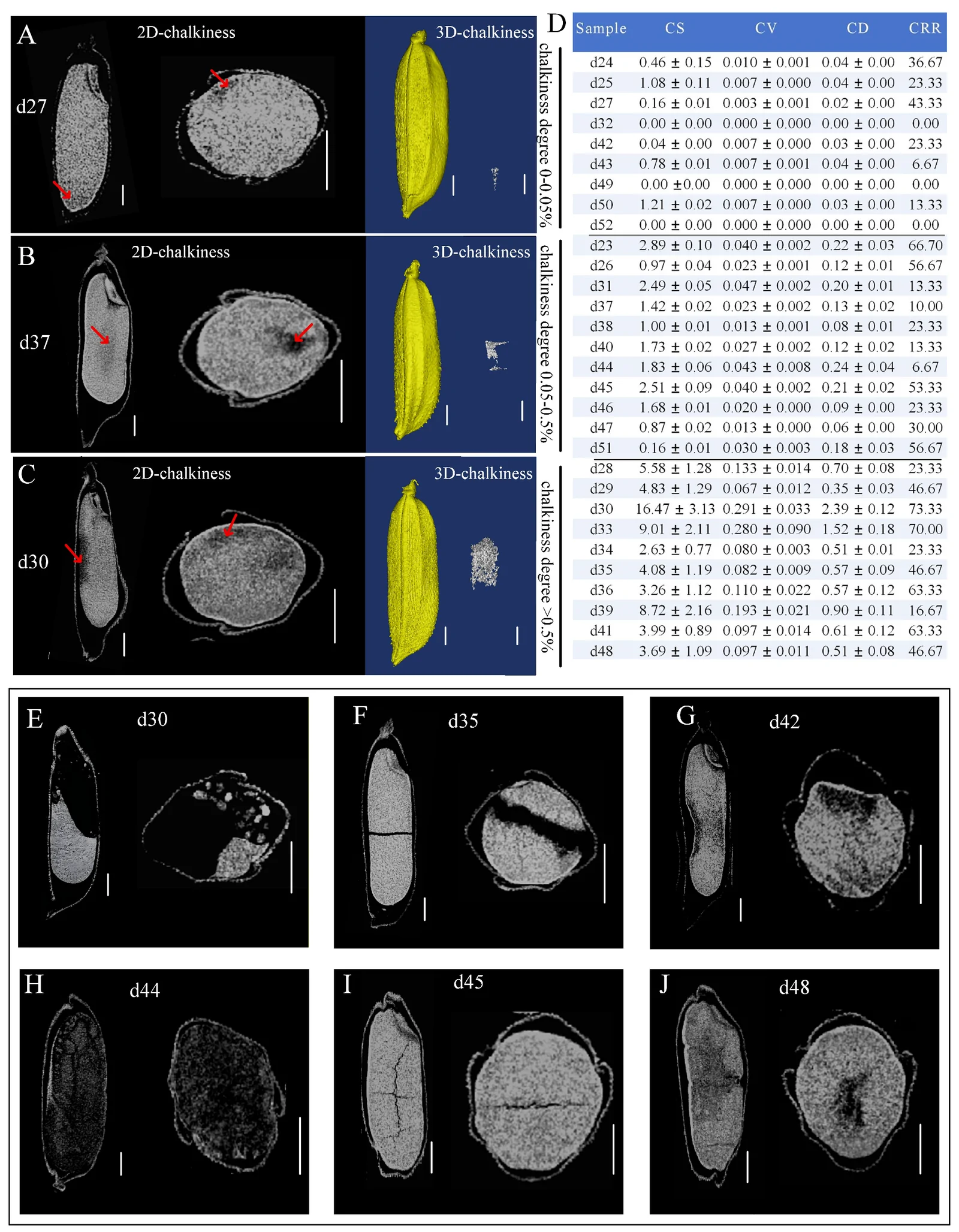
Visualization and quantification of appearance quality traits. Cross-section images (**left**) and 3D reconstructions (**right**) of representative varieties d27 (**A**), d37 (**B**) and d30 (**C**). Dark areas indicated by a red arrow represented the rice chalkiness. (**D**) Chalkiness in white rice grains. The cross-section images of endosperm hypoplasia and structural abnormalities of the kernels in d30 (**E**), d35 (**F**), d42 (**G**), d44 (**H**), d45 (**I**) and d48 (**J**). *n* = 30. Note: CS (mm^2^) and CV (mm^3^) represented the chalkiness surface area and chalkiness volume, respectively. CD (%) and CRR (%), respectively, represented the chalkiness degree and chalky rice rate. Internal defects analysis of white rice.

Building upon the established methodology based on μCT, we generated 3D reconstructions of internal structures and systematically assessed the representative appearance quality traits (chalkiness) across 30 white rice cultivars (Table S1). An array with 30 unhusked rice grains was scanned by X-ray under 12 μm of spatial resolution. The chalkiness areas and their borders were easily observed in cross-sections. The spatial location and shape of the cubic chalkiness part were visualized through the reconstructed 3D chalkiness image, and the chalkiness degree was calculated based on the volume data provided by the built-in software (Figure 2A). Beyond representative indicators of chalkiness (e.g., chalky rice rate and chalkiness degree), we also visualized its shape and obtained the grain surface area, grain volume, chalkiness surface area and chalkiness volume (Figure 2A–D).

The results showed that the chalkiness surface area of 30 varieties ranged from 0 to 16.47 mm^2^, chalkiness volumes from 0 to 0.29 mm^3^, chalky rice rate from 0 to 73.33%, and chalkiness degrees from 0 to 2.39%. The group with a 0–0.05% chalkiness degree (Figure 2A) included nine varieties (d24, d25, d27, d32, d42, d43, d49, d50, d52), and three varieties (d32, d49 and d52) have almost no chalkiness. The group with a 0.05–0.5% chalkiness degree (Figure 2B) contained eleven varieties (d23, d26, d31, d37, d38, d40, d44, d45, d46, d47, d51). Varieties with a chalkiness degree > 0.5% (Figure 2C) include ten varieties (d28, d29, d30, d33, d34, d35, d36, d39, d41, d48). Among these, the quality of d30 was relatively low, which had a chalkiness surface area of 16.47 mm^2^, volume of 0.29 mm^3^, chalkiness degree of 2.39%, and chalky rice rate of 73.33% (Figure 2A). Meanwhile, our micro-CT analysis successfully resolved critical structural anomalies within intact husks, providing a high-resolution 3D discrimination of defective grain features (Figure 2E–J). Unfilled kernels resulting from incomplete grain filling, such as sample d44 (Figure 2H), exhibited near-total internal voiding and characteristics of empty hulls. Endosperm hypoplasia was distinctly visualized in d42 (Figure 2G) and d48 (Figure 2J), where irregular gray–black patches indicated tissue collapse under development. Notably, pervasive micro-fissure in the endosperm of cultivars d35 (Figure 2F) and d45 (Figure 2I) manifested as linear black trajectories traversing the otherwise solid white endosperm matrix. Severe structural deficiency was observed in d30, which showed fragmentation across more than half of the kernel (Figure 2E). We believe that insect tunneling and pathogenic infection trajectories also can be visualized in rice grains. These defect differentiations underscore the capacity of μCT for the non-destructive triage of rice quality traits. In short, our micro-CT approach enabled a 3D quantification of rice appearance traits, such as the volume, morphology and spatial distribution in living rice grains. This non-destructive technique can also help to detect inaccessible subsurface defects.

### 2.3. Bran Layer and Embryo as Indirect Indicators of Nutrition Quality Can Be Determined

Except for the dominant starch in endosperm, the nutritional components that people are more concerned about are lipids, protein/amino acids, nucleic acids, vitamins, mineral elements, dietary fiber and other bioactive substances. In fact, these components mainly exist in embryos, and the composite layer consisting of pericarp, the seed coat and aleurone. The size of the embryo and thickness of the composite layer can indirectly reflect the nutrition quality. Consequently, the visualization and determination of the composite layer and embryo traits, including length, width, height, surface area and volume, are very helpful for intuitively assessing rice nutrition quality and accelerating the breeding of higher-quality rice.

**Figure 3:**
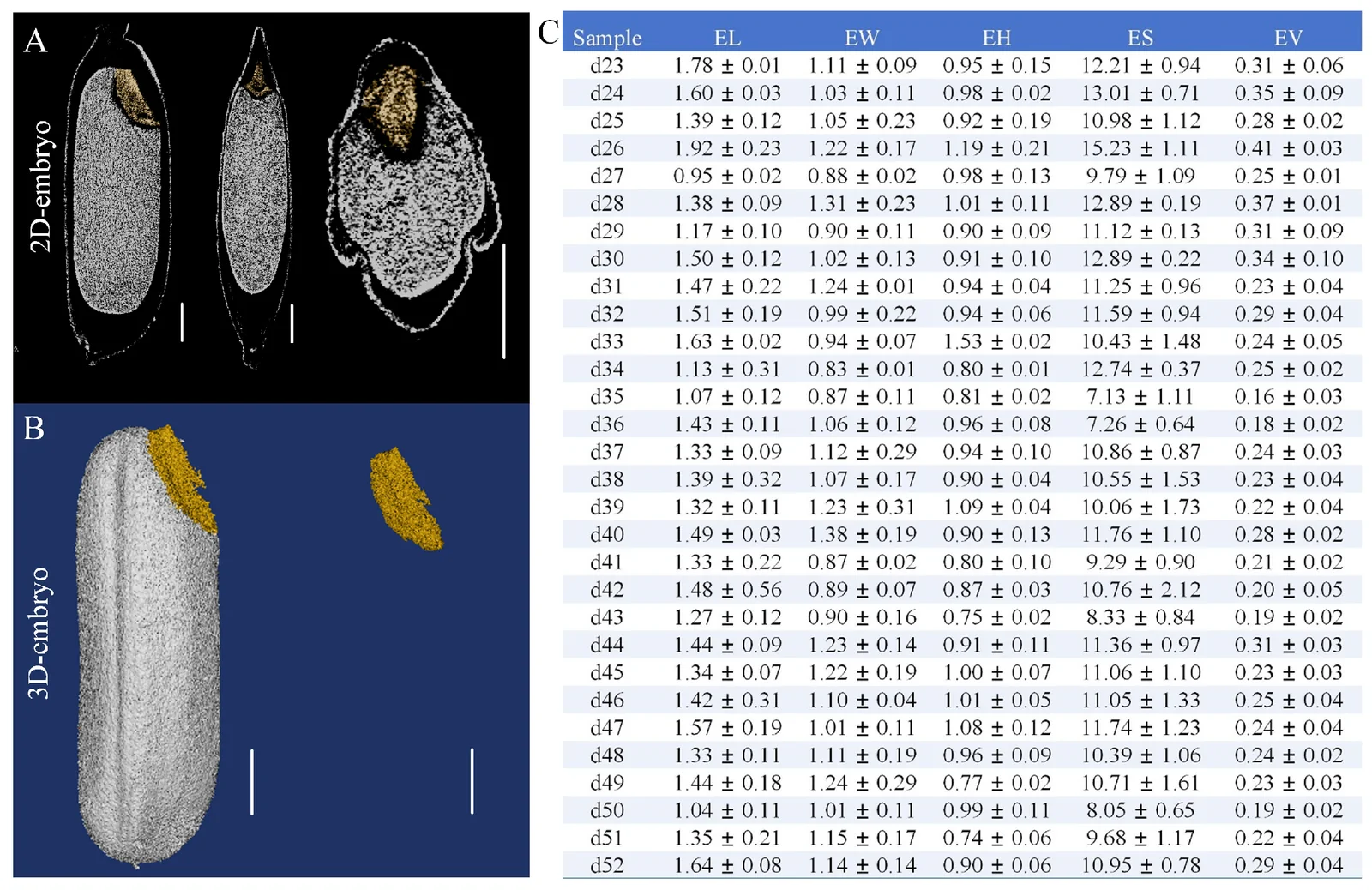
3D analysis of rice embryo. (**A**) The 2D cross-sections of rice embryo. (**B**) The 3D reconstruction of rice embryo. (**C**) Quantitative analysis of husk-enclosed grains and endosperm traits across 30 white rice varieties. Note: EL (mm), EW (mm), EH (mm), ES (mm^2^) and EV (mm^3^), respectively, represent the embryo length, embryo width, embryo height, embryo surface area and embryo volume.

In this study, the embryo dimensions (length, width, height, surface area, and volume) of 30 white rice varieties were robustly quantified via micro-CT imaging (Figure 3). This was achieved by segmenting the embryonic structures from the 2D cross-sections (Figure 3A) and subsequently performing 3D reconstruction, enabling the isolation of intact three-dimensional embryos (Figure 3B). The results showed that the embryo length ranged from 0.95 mm to 1.92 mm, width from 0.83 mm to 1.38 mm, and height from 0.74 mm to 1.53 mm (Figure 3C). The calculated surface area varied between 7.13 mm^2^ and 15.23 mm^2^, while the estimated embryo volume spanned from 0.16 mm^3^ to 0.41 mm^3^ (Figure 3C). Notably, variety d26 exhibited both the largest volume and surface area, whereas d35 possessed the smallest volume and surface area. A positive correlation was observed between grain dimensions and both surface area and volume with larger grains generally displaying higher values for these parameters.

**Figure 4:**
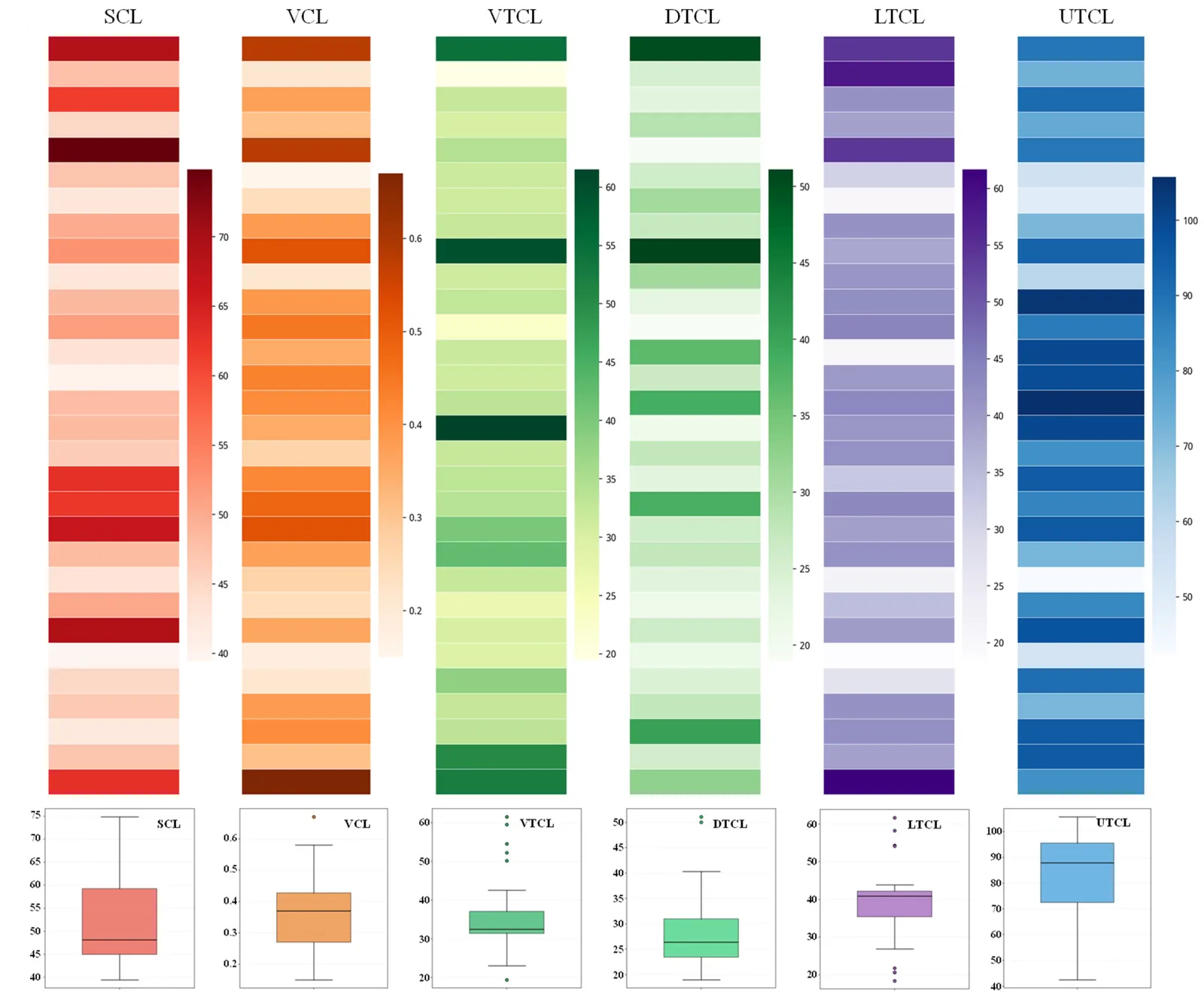
Qualification of the composite layer of pericarp, seed coat and aleurone. SCL: surface area of composite layer (mm^2^); VCL: volume of composite layer (mm^3^); VTCL: ventral thickness of composite layer (μm); DTCL: dorsal thickness of (μm) composite layer; UTCL: upper thickness of composite layer (μm); LTCL: lower thickness of composite layer (μm).

Meanwhile, segmentation of the bran layer was performed, enabling the calculation of the surface area and volume, as well as the measurement of thickness at the ventral, dorsal, upper, and lower regions (Figure 4, Table S2). The surface area of the bran layer ranged from 39.46 mm^2^ to 69.19 mm^2^, while the volume varied between 0.18 mm^3^ and 0.66 mm^3^. Regional thickness measurements revealed values of 19.41–61.50 μm at the ventral side, 18.97–50.03 μm at the dorsal side, 42.49–103.77 μm at the upper region, and 18.41–61.66 μm at the lower region. Notably, the thickness of the ventral, dorsal, and lower composite layers showed a relatively small variation among the varieties. In contrast, the upper composite layer was significantly thicker, exceeding the thickness of other regions by approximately two to four times. Among all varieties, d46 exhibited the largest composite layer in terms of both surface area and volume, whereas d47 displayed the smallest. These results highlight considerable inter-varietal differences in the composite layer volume, underscoring the potential of μCT as a powerful tool for the non-destructive phenotyping of internal structures associated with rice nutrition quality.

### 2.4. Nondestructive Identifying Quality Traits in Pigmented Rice

**Figure 5:**
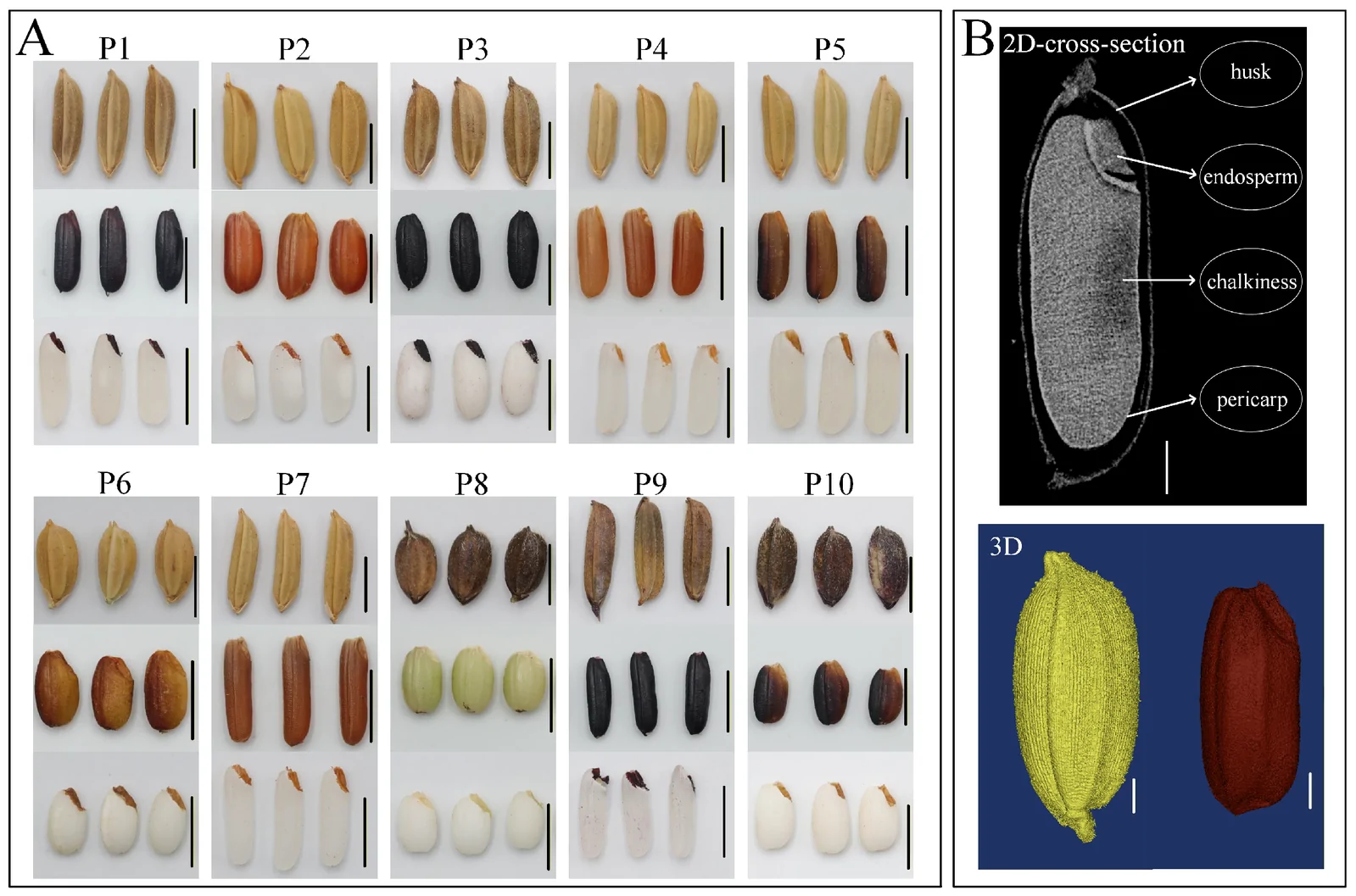
Nutrition quality analysis of 10 pigmented rice varieties. (**A**) Paddy grains, brown rice, and milled rice of P1–P10. (**B**) 2D cross-section images and 3D reconstruction images of pigmented rice obtained via micro-CT.

Pigmented (black, red and purple) rice is becoming increasingly popular for its rich bioactive compounds, such as proanthocyanidins, anthocyanins, flavonoids, phenolics and dietary fiber, which impart significant antioxidant capacities and health-promoting benefits. Studies confirm that these functional components are predominantly localized in the embryo and composite layer of the pericarp, seed coat and aleurone. Regretfully, the colored composite layer and embryo have become barriers for observing and detecting internal structures and appearance quality traits. When grading pigmented rice, some key quality traits such as chalkiness were not specifically examined. The methods in current rice standards cannot meet the requirements for a quality evaluation of pigmented rice. Here, we characterized 10 pigmented rice varieties, which contain a deep-hued husk and a colored composite layer in red, yellow, purple, green, and black, respectively (Figure 5A). After husking and milling, the milled rice kernels also displayed white endosperm. In order to comprehensively analyze the quality of pigmented rice, we captured the cross-sections of living rice grains by using micro-CT and performed segmentation and 3D reconstruction. Subsequently, some quality traits were visualized and determined in 2D and 3D (Figure 5B).

**Figure 6:**
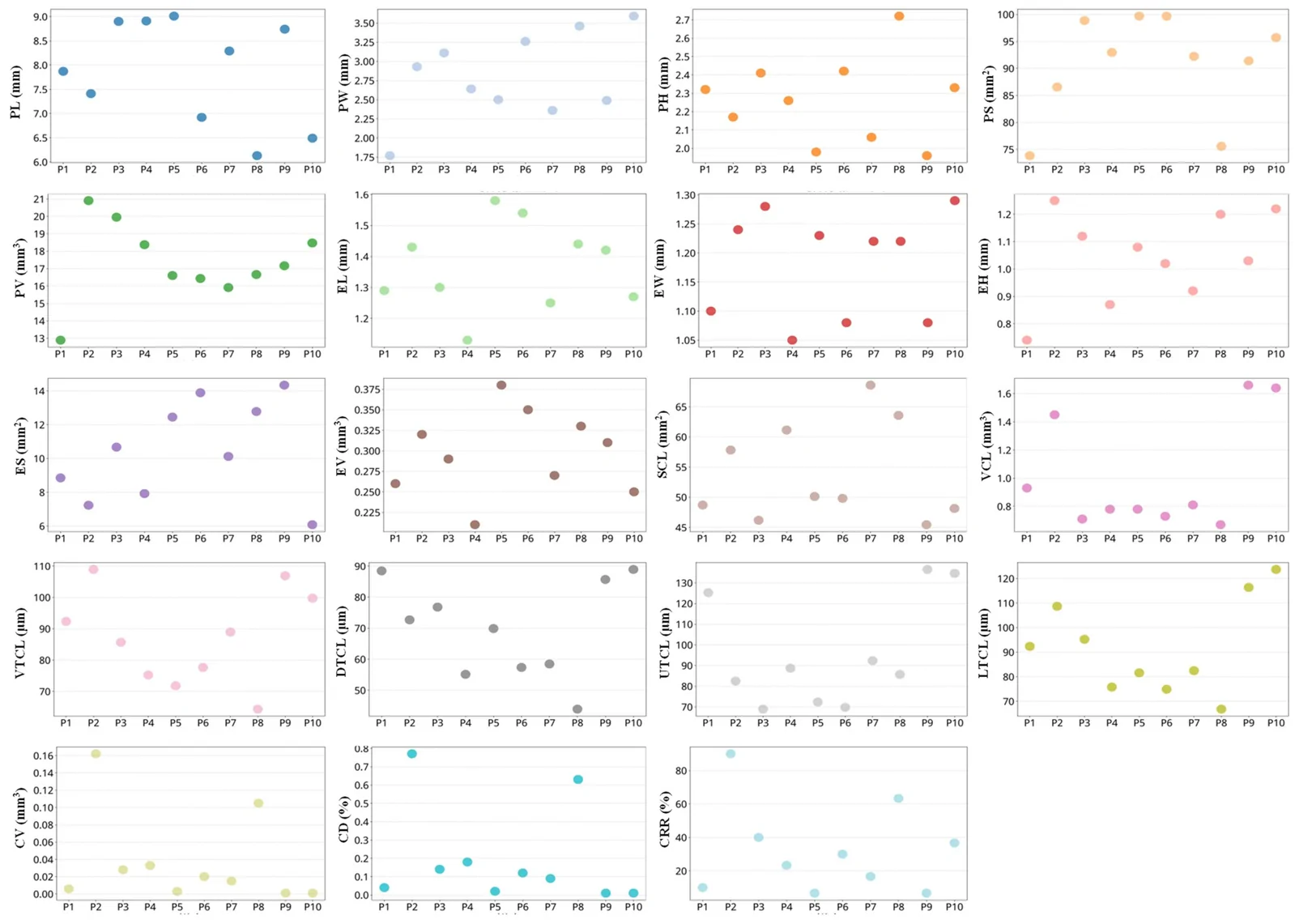
Quantitative analysis of quality traits in pigmented rice varieties. PL (mm), PW (mm), PH (mm), PS (mm^2^) and PV (mm^3^), respectively, represented the paddy grain length, paddy grain width, paddy grain height, paddy grain surface area and paddy grain volume. EL (mm), EW (mm), EH (mm), ES (mm^2^) and EV (mm^3^), respectively, represented the embryo length, embryo width, embryo height, embryo surface area and embryo volume. SCL (mm^2^), VCL (mm^3^), VTCL (μm), DTCL (μm), UTCL (μm) and LTCL (μm) represented the surface area, volume, ventral thickness, dorsal thickness, upper thickness and lower thickness of the composite layer, respectively. CV (mm^3^), CD (%) and CRR (%), respectively, represented the chalkiness volume, chalkiness degree and chalky rice rate.

The internal chalkiness was detected in all varieties via density thresholding (Figure 6, Table S3). The chalkiness volume, chalkiness degree, and chalky rice rate were in the range of 0.001–0.162 mm^3^, 0.01–0.77% and 6.67–90%, respectively. Notably, P2 exhibited higher chalkiness levels (0.162 mm^3^ of chalkiness volume, 0.77% of chalkiness degree and 90% of chalky rice rate). Conversely, P9 and P10 demonstrated minimal chalkiness (0.001 and 0.001 mm^3^ chalkiness volume, 0.01% and 0.01% of chalkiness degrees, 6.67% and 36.67% of chalky rice rate), demonstrating superior quality. Concurrently, the composite layer of the pericarp, seed coat and aleurone was segmented, and its surface area (45.45–68.57 mm^2^), volume (0.67–1.66 mm^3^) and thickness of the ventral (64.32–106.89 μm), dorsal (43.89–88.91 μm), upper (69.78–136.45 μm), and lower (66.78–123.67 μm) regions were evaluated. The composite layer volume of the majority of varieties remained at approximately 0.7 mm^3^ with only a few exceeding 1.0 mm^3^. In contrast, pigmented rice varieties exhibited significantly larger composite layer volume and thickness that was generally one to two times greater than those of white rice. Among the different regions (ventral, dorsal, upper, and lower) of the bran layer in colored rice, the upper region consistently showed greater thickness, while no notable differences were observed among the ventral, dorsal, and lower regions. Interestingly, the correlation was observed between the darkness of the pericarp color and the structural parameters of the composite layer: darker-colored varieties tended to have a larger composite layer volume, along with increased thickness in the ventral, dorsal, and lower regions. Conversely, lighter-colored varieties displayed smaller volume and reduced thickness. For instance, varieties P1, P9, and P10, with black or dark purple pericarps, had 0.93 mm^3^, 106.89 mm^3^ and 99.75 mm^3^ of composite layer volumes, respectively, while P8, the lightest-colored variety, only had a 0.67 mm^3^ volume. A larger thickness and volume means more nutritional components; thus, this method can be introduced to evaluate the nutrition quality of both white and pigmented rice. Our method solves the problem of the inability to visualize and quantify the color layer in pigmented rice, will promote the development of pigmented rice breeding, and can guide the identification of pigmented rice quality.

### 2.5. Phenotyping Other Traits in Living Rice Seeds

Optimal grain dimensions (length and width) and a higher percentage of whole milled kernels are crucial. Larger grains with a high length-to-width ratio contribute to higher head rice yield by reducing breakage during husking and milling, thereby minimizing nutrient loss and enhancing marketability. The measurement of grain size largely relies on conventional manual tools (such as vernier calipers or micrometers), which is not only time-consuming but also fails to capture the three-dimensional information. Traditional grain and kernel measurements predominantly quantify length and width parameters but overlook thickness, which is an important trait influencing milling yield and compaction. The kernel-to-hull volume ratio and the percentage of unfilled grains also cannot be quantified by using current methods.

Here micro-CT was employed to characterize the morphological attributes of paddy grains and kernels in 30 white rice varieties. Parameters included the length, width, height, surface area, and volume of paddy grains and volume of kernels. Notably, kernel dimensions were obtained without physical de-hulling. We instead applied a 2D segmentation and 3D reconstruction workflow to digitally isolate and model the kernels within their hulls. This non-destructive technique provided a direct measurement of the hull cavity volume and the rate of unfilled grains.

**Figure 7:**
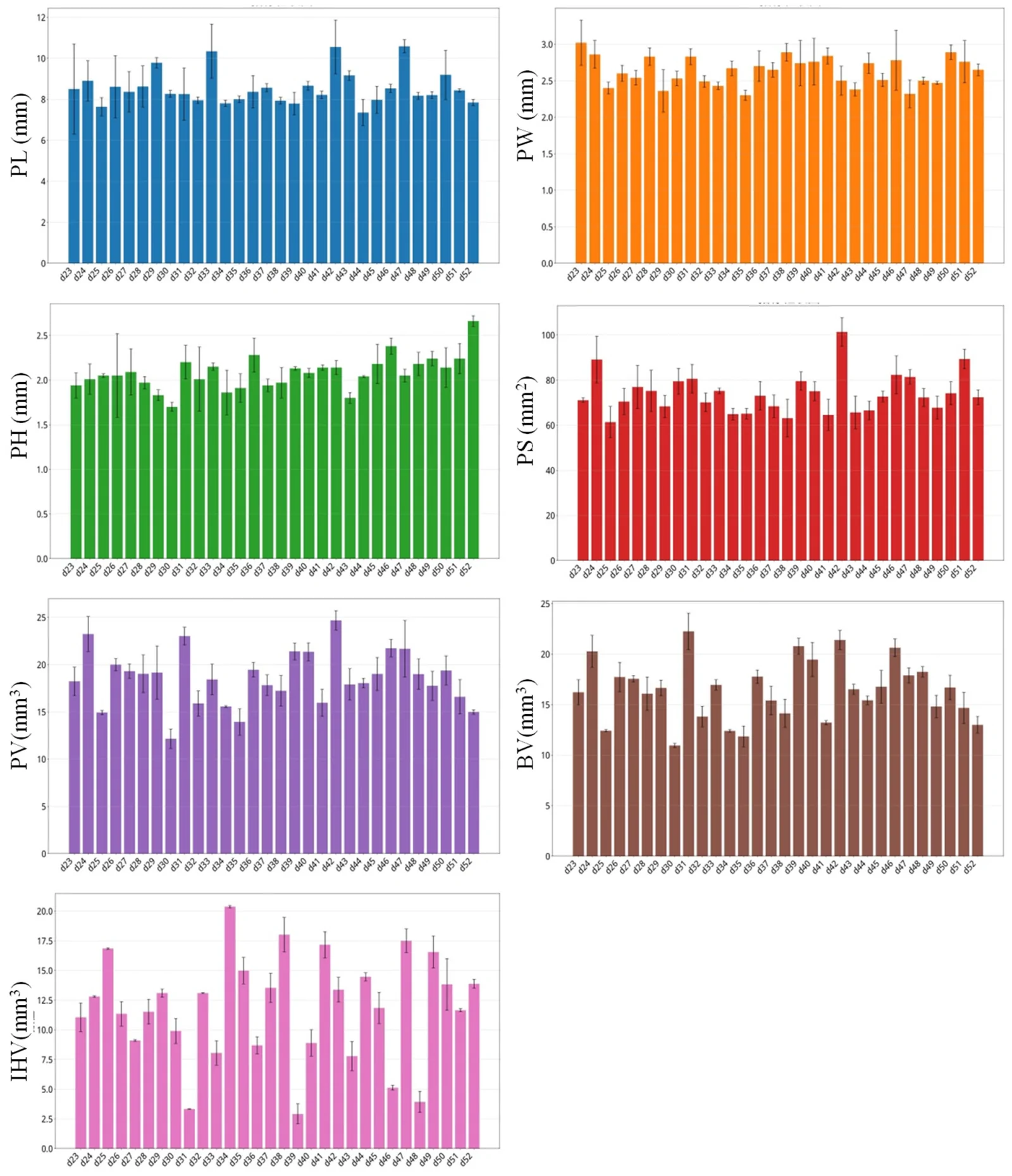
Phenotyping other traits in living paddy grains. Note: PL (mm), PW (mm), PH (mm), PS (mm^2^), PV (mm^3^), BV (mm^3^) and IHV (mm^3^), respectively, represent the paddy grain length, paddy grain width, paddy grain height, paddy grain surface area, paddy grain volume, brown rice volume and internal hollow volume.

The varieties exhibited significant differences in phenotypic traits (Figure 7, Table S4). The results showed that the 30 white rice varieties exhibited paddy grain dimensions ranging 7.35–10.58 mm (length), 2.30–3.02 mm (width), and 1.70–2.66 mm (thickness). The paddy grain surface area and volume ranged from 61.36 to 101.35 mm^2^ and 12.15 to 24.68 mm^3^, while the brown rice volume ranged from 10.95 to 22.24 mm^3^. The results revealed significant variation in the internal hollow volume among different rice varieties. Most varieties exhibited an internal hollow volume ratio of approximately 11–13%, while several distinctive outliers were observed. For instance, d34 showed a notably high internal hollow volume ratio (20.37%), whereas d39 had a considerably lower ratio (only about 2.91%). The low internal hollow volume ratio in d39 suggests well-developed and plump grains, which is considered a favorable trait for rice quality.

## 3. Discussion

Currently, people have higher demands for rice quality along with the improvement of living standards. Rice quality has become a decisive factor for rice production, consumption and circulation [3,4]. The quality of rice is mainly evaluated from several aspects such as milling, appearance, nutrition, eating, cooking and physicochemical characteristics [4,6]. Among them, milling, appearance and nutrition qualities largely determine the market value and consumer preference [7]. In recent years, people have paid special attention to the nutrition of rice rather than its calories. The nutritional components of consumer preference include protein/amino acids, nucleic acids, vitamins, mineral elements, dietary fiber and other bioactive substances. Regretfully, these nutritional components have been almost completely removed in well-milled rice, since these components mainly exist in embryos and the composite layer consisting of the pericarp, seed coat and aleurone. Brown (whole) rice and pigmented rice with a preserved embryo and the composite layer are thereby becoming increasingly popular [7,43]. In recent years, rice quality improvement has become one of the most important goals for crop scientists and breeders. Great progress has been made in understanding the genetic and molecular mechanisms underlying quality traits [10,44,45]. Some important genes associated with rice quality have been identified, such as *Chalk5* [44], *FLO2* [46], *FLO7* [47], *OsPPDKB* [48], and *OsRab5a* [49].

As is well known, methodology is the key to promoting scientific and technological progress. In the field of rice quality assessment and research, the detection methods still face significant limitations and challenges. According to the current rice standards, the visual inspection (naked-eye observation) and projection image-based approaches are still mainstreams in rice quality evaluation. Projection image-based methods misinterpret overlapping and hiding structures, and they fail to determine true 3D traits, such as the 3D shape, subsurface, location and volume. These methods also overlook the detection of inner defects, such as fissures, insects, lesions caused by pathogenic microorganisms and incompleteness. Some chemical methods are employed to detect the contents of nutritional components and the physicochemical characteristics, but the related processes are complex and time-consuming. The husking/milling process recommended in the rice standards is destructive, and the rice embryo, pericarp, seed coat and aleurone layer are completely removed. Current methods based on milled rice cannot be fully applicable to phenotype the inner traits of living rice seeds. In addition, people implement different standards when evaluating pigmented rice and white rice. In this study, we employed microCT to capture a series of continuous cross-sections of living (white/pigmented) rice seeds. After performing segmentation and reconstruction, we simultaneously visualized and quantified multiple rice quality traits hidden in living white/pigmented rice seeds both at the 2D and 3D level. Through one group of cross-section images (DCM format) and in silico analysis, we obtained the 2D indicators, such as the width, length, thickness, shape and projection area. After 3D reconstructing, it allowed for calculating the cubic information (shape, location, surface area and volume) of critical quality traits, such as the chalkiness, embryo, endosperm, composite layer (pericarp, seed coat and aleurone), and defects. Comparing with HIS, NIR, FTIR and EM, our approach also shows obvious advantages in the terms of accuracy, efficiency, cost and throughput, although the cost of the micro-CT instrument is relatively high (Table 3). Accordingly, this method overcomes the limitations of current approaches of rice quality evaluation, improves the detection efficiency and scope, and is expected to become a new testing standard of rice quality. It will become a strong technical support for accelerating the genetic breeding of high-quality rice. It should be noted that these microCT-derived structural parameters are indirect proxies for nutritional quality; their quantitative correlations with actual nutrient contents were not determined in this study and require further chemical validation.

## 4. Materials and Methods

### 4.1. Sample Collection and Preparation

A total of 40 distinct rice varieties were used in this study, comprising 30 white rice cultivars and 10 pigmented rice varieties (Supplemental Table S1). Shanlan germplasms are upland rice cultivated in Baoting Li-Miao autonomous county (18°25′ N, 109°26′ E) of Hainan province. Other pigmented rice varieties were collected from Hainan province by our group. White rice seeds were collected from the Rice Germplasm Resource Bank of Hunan Province. Brown rice (30 grains per variety) was prepared by removing the husk using a Mini De-husker (Taizhou Cereal Instrument Co., Ltd., Taizhou, China), and the milled rice (30 grains per variety) was prepared by a mini milling machine (LTJM160, Taizhou Cereal Instrument Co., Ltd., Taizhou, China). For each variety, 30 living paddy grains were used as biological replicates (*n* = 30 grains per variety) and embedded in Super Light Clay (Alibaba, Hangzhou, China) at regular intervals to form one array (3 × 5 × 2). After solidification, a cubic sample (approximately 2 cm × 2 cm × 2 cm) containing the 30 grains was prepared and subjected to one micro-CT scan at 12 μm spatial resolution. Thus, 40 micro-CT scans were performed in total (one per variety), and 1200 grains were analyzed. No separate technical replicates were performed; all 2D and 3D traits were derived from the same scan dataset of the same grains. Measurements were first obtained at the individual grain level and then averaged within each variety; therefore, the individual grain was the experimental unit (*n* = 30 per variety), and variety-level means ± SD are reported.

### 4.2. X-Ray Scanning and μCT Device Setting

Each sample was loaded into µCT 100 (SCANCO Medical AG, Brüttisellen, Switzerland) containing a cabinet cone-beam, sealed, air-cooled X-ray source with 5–30 µm spot size and 1.25–200 µm nominal isotropic of resolution. The samples were positioned at proper distances from the X-ray source according to the sample size and spatial resolution, representing the distance between adjacent cross-section images. The micro-CT operation included resolution setting, current and voltage setting, program running and data saving according to the operation manual. The sample can be rotated in the sample chamber (a glass tube) and scanned in three dimensions. The spatial resolution, voltage and electric current were set at 12 μm, 50 kV and 120μA, respectively. The signals of the transmitted X-ray were captured and imaged by an X-ray camera. The cross-sections were exported as DCM format files containing actual size information, which is more compatible with the subsequent segment and analysis software.

### 4.3. Measurement and View of Size and Area-Related Indicators in Cross-Section

Referring to our previous method [42], the DCM files of each sample were imported into Mimics software19.0 in the format of strict DICOM 3.0 (digital imaging and communications in medicine 3.0). A series of monochrome cross-section images was displayed in three views (top–bottom, right–left and front–back). The size-related indicators/trait in 2D, including length/width and thickness, were directly measured in cross-sections by measurement tools. The area value of the cross-sections could be directly viewed.

### 4.4. Segmentation and 3D Reconstruction of Structural Components in Rice Grain

According to our previous work [42], three views of cross-sections were imaged in processing software Mimics. Through the tools of “Thresholding”, “Contrast”, “Region Growing”, “Edit Masks”, and “Morphology Operation”, the structural components in the rice grain were segmented from original cross-sections to obtain new masks (parts of cross-sections). Each group of masks represented a rice quality trait. The 2D size-related indicators and area value of a quality trait were also measured and directly read by the related tools. By using “Calculate 3D”, a group of masks was reconstructed as a 3D object, which is a cubic trait. The reconstructed 3D trait was showed in a window and allowed free rotation and viewing. The values of the surface area and volume could be viewed by using “3D Properties”. Notably, the surface area of the embryo and endosperm includes both their external surfaces and their contact interfaces with adjacent tissues rather than solely the exposed outer biological surfaces. A unified step-by-step segmentation and 3D reconstruction workflow is provided in Figure S1.

In this study, the husk, kernel, endosperm, embryo, chalkiness, outer layer of brown rice (the composite layer composed of the pericarp, seed coat and aleurone), defects (fissure, insect damage, pathogenic microorganism invasion and failed filling) and hollow traits were visualized and quantified. The percentage of rice chalkiness was calculated. The internal hollow volume ratio (%) was calculated as (paddy grain volume − brown rice volume)/paddy grain volume × 100%. The chalkiness degree (3D) was calculated as (chalkiness volume/paddy grain volume) × 100%. The internal hollow volume ratio (%) was calculated as (paddy grain volume − brown rice volume)/paddy grain volume × 100%.

### 4.5. Computer Setting

Processing software was running in a desktop computer powered by 64 bit Windows 11 (Microsoft, New York, NY, USA). To favorably perform a 3D reconstruction of rice grains, the optimal hardware configuration included an Intel Core i7-9750H CPU (64 bit), 64 gigabytes of memory and a GeForce RTX 2060 graphics card (NVIDIA, Santa Clara, CA, USA).

### 4.6. Data Processing and Statistics

For each variety, 30 living grains/kernels were used as biological replicates, and each grain/kernel was treated as the experimental unit. All microCT-derived measurements were obtained in Mimics and exported to Microsoft Excel for summary statistics. Data are presented as mean ± standard deviation (SD) and range. Variety-level differences and trait relationships were described using summary statistics, box plots and scatter plots. The number of replicates (*n* = 30 grains per variety) is indicated in the figure legends. All analyses were performed using Microsoft Excel and GraphPad Prism 9.0.

## 5. Conclusions

In this study, we employed microCT to scan a living seed matrix and captured the cross-section images of 30 white rice cultivars and 10 pigmented rice varieties. A workflow for the visualization and determination of quality traits was constructed. We simultaneously determined multiple quality traits hidden in living white/pigmented rice seeds both at the 2D and 3D level after contrasting and segmenting by using one group of cross-section images from one scanning. Almost all appearance traits can be detected at the 2D and 3D level. More quality traits and other structural traits can be non-destructively determined both in white and pigmented rice. Although these structural traits may serve as indirect indicators of nutritional quality, their correlations with actual nutrient contents were not quantified here and should be interpreted as tentative until chemically validated. This research also expanded the scope of quality-related traits, displayed their actual 2D- and (or) 3D information, and improved the capability and efficiency. The achievements can guide the establishment of a new unified standard for white and pigmented rice at the 3D level. This multi-trait quantification capability within a single and rapid scan underscores its greatly applicable potential in rice quality assessment, germplasm screening and genetic breeding.

## Figures and Tables

**Table 1:** Major accessed quality traits and testing method in executing rice standards.

	Traits	Preprocessing	Recommended Method in Executing Standard	Quality Type
White rice	Chalkiness	Husking and milling: all of the bran and embryo have been removed	Calculating projected area (2D)	Milling and appearance
	Size		Measurement using a ruler and calculating in the projection diagram	Appearance
	Broken kernel		Counting by hand or computer	Milling and appearance
	Defective/damaged kernel		Naked-eye observation and counting	Milling and appearance
	Moisture content		Dry weighing method	-
	Sensory score		Observation, smelling and tasting	Eating
	Amylose content	Powdering milled rice	Spectrophotometry	Nutrition
Pigmented rice	Moisture content	Husking	Dry weighing method	-
	Size		Measurement using a ruler and calculating in the projection diagram	Appearance
	Broken kernel		Counting by hand or computer	Milling and appearance
	Defective/damaged kernel		Naked-eye observation and counting	Milling and appearance
	Anthocyanin content	Powdering husked rice	Spectrophotometry	Nutrition

**Table 2:** Comparison of this method and executing standard method.

Points	This Study	Standard Method
Dimension	Both 2D and 3D	2D
Spatial resolution	μm, μm^2^, μm^3^	mm, mm^2^
Material	Unified methods for white and pigmented rice	Independent methods for white and pigmented rice
Bioactivity retention	Living paddy grains are used for in vivo analysis	Milling for white rice and husking for pigmented rice lead to the loss of seed vitality
Operation	Only one micro-CT scan followed by in silico analysis of multiple structural traits	Involving a series of preprocessing and testing steps
Outer layer of husked rice	Analysis of size, shape, thickness, surface area and volume; Indirectly reflecting nutrition quality	Not involved in white rice; anthocyanin content quantification in pigmented rice
Embryo	Analysis of size, shape, location, surface area and volume; Indirectly reflecting nutrition quality	Not involved
Chalkiness	Analysis of size, shape, location, surface area and volume	Undetectable in pigmented rice
Grain plumpness	Detectable	Undetectable
Grain fissure	Detectable	Undetectable
Wormy kernel	Detectable	Undetectable
Other traits	Awn, lemma, burr, palea, sterile lemma, and inner hollow	-

**Table 3:** Comparison of major methods for the analysis of rice quality traits.

Feature	This Method	HSI	NIR	FTIR	EM
Internal structure visualization	Yes	No	No	No	No
Sample preparation	None	Minimal	Minimal	Minimal	Extensive
Throughput	High	High	High	Moderate	Low
Relative cost	Low	Moderate	Low	Low	Moderate
Accuracy	High	Moderate	Moderate	Moderate	High
Efficiency	Fast	Fast	Fast	Fast	Slow

Note: HSI, hyperspectral imaging; NIR, near-infrared spectroscopy; FTIR, Fourier-transform infrared spectroscopy; EM, electron microscopy.

## Data Availability

The original contributions presented in this study are included in the article/supplementary material. Further inquiries can be directed to the corresponding authors.

## Supplementary Materials

The following supporting information can be downloaded at https://www.mdpi.com/article/10.3390/plants15203123/s1, Figure S1: Stepwise workflow of image import, semi-automatic segmentation, mask refinement, 3D reconstruction, and 2D/3D measurement used for rice quality trait quantification. Table S1: Rice varieties in this study. Table S2: Qualification of the composite layer of pericarp, seed coat and aleurone. Table S3: Quantitative analysis of quality traits in pigmented rice varieties. Table S4: Paddy grain traits in 30 white rice cultivars.
