# Supplementary material for: Simultaneous Quantification of Multiple Quality Traits Hidden in Living White/Pigmented Rice Seeds Both at 2D and 3D Level
Source: Plants (Basel). 2026 Oct 13;15(20):3123. doi: 10.3390/plants15203123 (PMC13708598; doi:10.3390/plants15203123)
Supplement: Supplementary file 1 [file plants-15-03123-s001.zip › plants-4494303-supplementary.pdf]

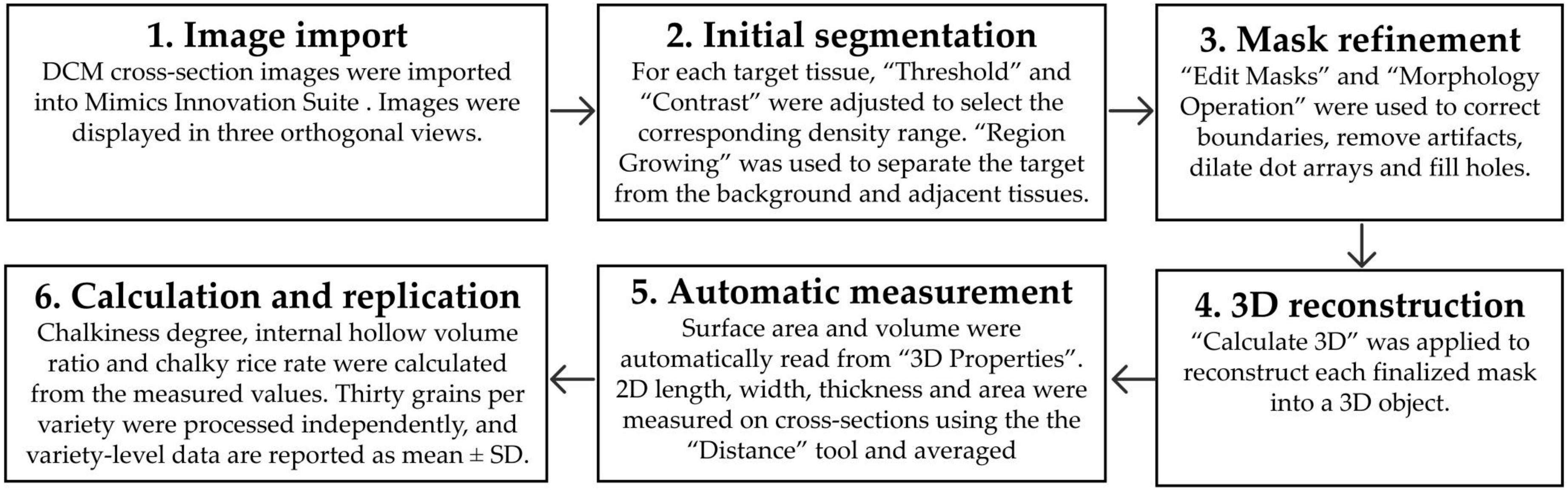

Note: “Threshold selection“, “Region Growing“, “Edit Masks“ and “Morphology Operation“ are semi-automatic and operator-dependent; “Calculate 3D“ and “3D Properties“ are automatic. The same workflow and the same threshold range for each tissue type were used across all varieties.

Figure S1 Stepwise workflow of image import, semi-automatic segmentation, mask refinement, 3D reconstruction, and 2D/3D measurement used for rice quality trait quantification.

Table S1 Rice varieties in this study

**No.**	Cultivar	Husk color	Kernel color	Feature	Subspecies
P1	shanlan (black)	yellow	black	upland rice	Indica
P2	shanlan (red)	yellow	red	upland rice	Japonica
P3	rice landrace (black)	yellow	black	conventional rice	Japonica
P4	rice landrace (red)	yellow	red	conventional rice	Indica
P5	rice landrace (purple)	yellow	purple	conventional rice	Indica
P6	rice landrace (yellow)	yellow	red	conventional rice	Japonica
P7	rice germplasm (red)	yellow	red	conventional rice	Indica
P8	rice landrace (green)	purple	green	conventional rice	Japonica
P9	purple-leaf black rice	yellow	black	conventional rice	Indica
P10	purple-leaf blood glutinous rice	purple	purple black	conventional rice	Japonica
d23	heng feng you zhi 903	yellow	white	conventional rice	Indica

d24	quan you 1114	yellow	white	conventional rice	Indica
d25	zhong xiang you 378 pu	yellow	white	conventional rice	Indica
d26	sui liang you 2199	yellow	white	conventional rice	Indica
d27	deng liang you 1898	yellow	white	conventional rice	Indica
d28	chun liang you nong 39	yellow	white	conventional rice	Indica
d29	zhen liang you 8439 you 3	yellow	white	conventional rice	Indica
d30	liang you 1908	yellow	white	conventional rice	Indica
d31	quan you 851 you 3	yellow	white	conventional rice	Indica
d32	min kai you si zhan you 3	yellow	white	conventional rice	Indica
d33	nong xiang 39 you 1	yellow	white	conventional rice	Indica
d34	qing xiang you 238	yellow	white	conventional rice	Indica
d35	guo tai xiang chun si you 3	yellow	white	conventional rice	Indica
d36	lian you hua zhan	yellow	white	conventional rice	Indica
d37	yue liang you 8656 you 3	yellow	white	conventional rice	Indica
d38	yun liang you 2935 you 3	yellow	white	conventional rice	Indica
d39	min kai you 4185 you 3	yellow	white	conventional rice	Indica
d40	7096 you zhi 903 you 3	yellow	white	conventional rice	Indica
d41	zhen liang you 3287 you 3	yellow	white	conventional rice	Indica
d42	yu jing 91 you 1	yellow	white	conventional rice	Indica
d43	hong liang you 503 you 2	yellow	white	conventional rice	Indica
d44	jing liang you 7818 you 3	yellow	white	conventional rice	Indica
d45	chuang yu 9hao you 2	yellow	white	conventional rice	Indica
d46	feng liang you si hao	yellow	white	conventional rice	Indica
d47	yu zhen xiang you 1	yellow	white	conventional rice	Indica
d48	zhen liang you 70122 you 2	yellow	white	conventional rice	Indica
d49	quan tai you yin tai xiang zhan you 3	yellow	white	conventional rice	Indica
d50	nong xiang 42 you 1	yellow	white	conventional rice	Indica
d51	xiang wan can 13hao you 2	yellow	white	conventional rice	Indica
d52	long jing you 1212 you 2	yellow	white	conventional rice	Indica

Table S2 Qualification of the composite layer of pericarp, seed coat and aleurone

Sample	Surface-area (mm^2)	Volume (mm^3)	Thickness (μm)			
			ventral	dorsal	upper	lower
d23	68.66 ± 0.19	0.58 ± 0.11	54.55 ± 1.01	50.03 ± 2.02	88.89 ± 13.20	54.30 ± 9.33
d24	47.71 ± 2.79	0.21 ± 0.07	19.41 ± 1.54	25.03 ± 6.15	73.31 ± 7.24	58.22 ± 5.95
d25	61.48 ± 1.55	0.37 ± 0.09	32.09 ± 8.33	23.33 ± 0.65	91.58 ± 12.20	41.45 ± 1.50
d26	44.98 ± 8.33	0.31 ± 0.08	30.17 ± 5.72	28.77 ± 4.25	75.57 ± 11.50	38.92 ± 5.98
d27	74.85 ± 9.80	0.58 ± 0.12	34.23 ± 5.09	19.11 ± 8.01	88.22 ± 23.00	54.10 ± 6.47
d28	47.23 ± 2.83	0.15 ± 0.02	31.50 ± 0.29	26.09 ± 1.53	55.32 ± 3.01	30.77 ± 8.10
d29	42.72 ± 3.16	0.24 ± 0.01	31.40 ± 2.18	30.85 ± 1.54	49.66 ± 10.00	20.63 ± 0.20
d30	50.07 ± 3.71	0.38 ± 0.09	32.09 ± 4.31	27.04 ± 0.67	71.63 ± 12.20	41.41 ± 8.99
d31	52.66 ± 9.90	0.52 ± 0.17	59.48 ± 8.54	51.11 ± 4.14	93.37 ± 13.20	38.02 ± 7.09
d32	42.65 ± 0.16	0.21 ± 0.02	31.39 ± 3.22	31.01 ± 9.54	60.54 ± 0.91	40.95 ± 2.20
d33	48.59 ± 9.30	0.39 ± 0.09	32.75 ± 1.58	22.93 ± 9.78	103.77 ± 8.50	42.22 ± 5.59
d34	51.48 ± 3.81	0.45 ± 0.11	23.03 ± 6.95	18.97 ± 1.02	87.54 ± 0.91	43.87 ± 9.29
d35	43.59 ± 3.23	0.35 ± 0.08	31.89 ± 2.11	36.87 ± 2.53	99.78 ± 12.30	20.66 ± 0.05
d36	40.08 ± 2.97	0.43 ± 0.03	31.11 ± 1.07	26.54 ± 0.43	98.74 ± 11.20	40.21 ± 3.29
d37	48.14 ±0.56	0.41 ± 0.05	33.30 ± 1.74	38.60 ± 3.83	105.73 ± 2.98	43.10 ± 8.93
d38	48.37 ± 3.58	0.35 ± 0.09	61.50 ± 9.23	20.93 ± 1.55	100.01 ± 7.34	40.74 ± 0.23
d39	46.29 ± 3.43	0.27 ± 0.09	32.20 ± 4.49	27.53 ± 3.69	82.20 ± 12.33	41.68 ± 8.09
d40	62.95 ± 3.11	0.42 ± 0.11	33.16 ± 2.74	23.38 ± 3.80	95.35 ± 5.77	32.91 ± 5.05
d41	62.00 ± 3.85	0.48 ± 0.18	33.88 ± 5.05	38.36 ± 4.81	85.30 ± 12.70	42.94 ± 8.44
d42	66.72 ± 1.20	0.52 ± 0.08	40.20 ± 3.09	25.84 ± 6.24	95.83 ± 13.54	39.06 ± 3.40
d43	48.22 ± 3.57	0.37 ± 0.09	42.55 ± 4.44	27.55 ± 2.66	72.20 ± 3.33	41.66 ± 0.39
d44	43.42 ± 3.22	0.27 ± 0.09	32.39 ± 9.42	23.59 ± 6.62	42.49 ± 12.38	21.74 ± 6.29
d45	50.47 ± 0.74	0.24 ± 0.06	26.70 ± 3.69	20.89 ± 9.54	84.63 ± 10.21	34.53 ± 7.58
d46	69.19 ± 4.97	0.36 ± 0.11	30.31 ± 4.94	26.28 ± 2.33	97.42 ± 9.72	39.79 ± 8.24
d47	39.46 ± 2.55	0.18 ± 0.07	29.09 ± 0.66	21.37 ± 6.14	54.22 ± 11.33	18.41 ± 2.09
d48	45.07 ± 1.66	0.21 ± 0.02	38.07 ± 5.21	24.32 ± 0.99	90.48 ± 20.90	26.88 ± 7.11
d49	46.67 ± 3.46	0.38 ± 0.09	32.51 ± 0.31	27.43 ± 1.66	71.82 ± 9.20	41.54 ± 0.28
d50	42.10 ± 3.12	0.41 ± 0.10	33.23 ± 1.74	40.31 ± 6.88	95.33 ± 12.79	42.00 ± 8.39
d51	47.13 ± 3.49	0.31 ± 0.02	50.19 ± 8.73	25.70 ± 0.24	95.57 ± 1.53	38.91 ± 1.05
d52	62.85 ± 5.91	0.67 ± 0.09	52.21 ± 10.49	32.55 ± 6.61	82.22 ± 2.33	61.66 ± 6.33

SCL: surface-area of composite layer (mm^2); VCL: volume of composite layer (mm^3); VTCL: ventral thickness of composite layer (μm); DTCL: dorsal thickness of (μm) composite layer; UTCL: upper thickness of composite layer (μm); LTCL: lower thickness of composite layer (μm).

Table S3 Quantitative analysis of quality traits in pigmented rice varieties

Sample	P1	P2	P3	P4	P5	P6	P7	P8	P9	P10
PL	7.87 ± 0.18	7.41 ± 0.02	8.90 ± 0.25	8.91 ± 0.19	9.01 ± 0.37	6.92 ± 0.16	8.29 ± 0.21	6.13 ± 0.14	8.74 ± 0.23	6.49 ± 0.17
PW	1.77 ± 0.03	2.93 ± 0.11	3.11 ± 0.13	2.64 ± 0.09	2.50 ± 0.08	3.26 ± 0.15	2.36 ± 0.07	3.46 ± 0.16	2.49 ± 0.06	3.59 ± 0.14
PH	2.32 ± 0.08	2.17 ± 0.06	2.41 ± 0.10	2.26 ± 0.07	1.98 ± 0.05	2.42 ± 0.11	2.06 ± 0.04	2.72 ± 0.12	1.96 ± 0.03	2.33 ± 0.09
PS	73.83 ± 10.73	86.54 ± 2.38	98.85 ± 7.91	92.95 ± 11.22	99.68 ± 5.09	99.66 ± 9.32	92.22 ± 1.12	75.55 ± 2.81	91.39 ± 5.38	95.69 ± 4.11
PV	12.89 ± 3.01	20.90 ± 1.32	19.95 ± 4.01	18.37 ± 5.91	16.6 ± 2.33	16.43 ± 3.21	15.91 ± 1.76	16.66 ± 2.38	17.16 ± 2.21	18.47 ± 1.98
EL	1.29 ± 0.25	1.43 ± 0.37	1.30 ± 0.14	1.13 ± 0.13	1.58 ± 0.09	1.54 ± 0.28	1.25 ± 0.07	1.44 ± 0.16	1.42 ± 0.15	1.27 ± 0.04
EW	1.10 ± 0.14	1.24 ± 0.05	1.28 ± 0.16	1.05 ± 0.08	1.23 ± 0.25	1.08 ± 0.13	1.22 ± 0.34	1.22 ± 0.09	1.08 ± 0.12	1.29 ± 0.36
EH	0.74 ± 0.13	1.25 ± 0.28	1.12 ± 0.35	0.87 ± 0.02	1.08 ± 0.14	1.02 ± 0.09	0.92 ± 0.19	1.20 ± 0.37	1.03 ± 0.13	1.22 ± 0.36
ES	8.85 ± 1.55	7.23 ± 1.23	10.67 ± 0.67	7.92 ± 0.76	12.45 ± 1.02	13.89 ± 0.95	10.12 ± 0.83	12.78 ± 0.97	14.34 ± 0.21	6.08 ± 0.15
EV	0.26 ± 0.01	0.32 ± 0.11	0.29 ± 0.04	0.21 ± 0.01	0.38 ± 0.06	0.35 ± 0.05	0.27 ± 0.04	0.33 ± 0.10	0.31 ± 0.09	0.25 ± 0.03
CLS	48.70 ± 2.33	57.82 ± 5.07	46.18 ± 3.45	61.13 ± 9.21	50.12 ± 2.11	49.81 ± 1.34	68.57 ± 7.09	63.56 ± 5.03	45.45 ± 1.77	48.14 ± 3.98
CLV	0.93 ± 0.03	1.45 ± 0.33	0.71 ± 0.15	0.78 ± 0.19	0.78 ± 0.02	0.73 ± 0.02	0.81 ± 0.21	0.67 ± 0.11	1.66 ± 0.03	1.64 ± 0.29
TCV	92.34 ± 3.21	108.92 ± 9.87	85.67 ± 4.12	75.23 ± 6.34	71.78 ± 1.45	77.65 ± 3.56	88.91 ± 5.67	64.32 ± 4.23	106.89 ± 9.78	99.75 ± 3.89
TCD	88.45 ± 1.89	72.67 ± 3.45	76.78 ± 2.34	55.12 ± 4.56	69.87 ± 1.67	57.34 ± 2.12	58.45 ± 3.78	43.89 ± 2.97	85.67 ± 1.45	88.91 ± 2.67
TCU	125.23 ± 8.45	82.45 ± 9.67	68.90 ± 7.23	88.67 ± 11.23	72.34 ± 6.78	69.78 ± 5.89	92.34 ± 10.45	85.67 ± 8.90	136.45 ± 15.67	134.56 ± 11.89
TCL	92.34 ± 3.45	108.67 ± 2.89	95.23 ± 4.12	75.78 ± 3.67	81.56 ± 2.34	74.89 ± 3.78	82.45 ± 2.67	66.78 ± 2.45	116.34 ± 13.89	123.67 ± 13.23
CV	0.006 ± 0.000	0.162 ± 0.077	0.028 ± 0.010	0.033 ± 0.012	0.003 ± 0.000	0.020 ± 0.009	0.015 ± 0.004	0.105 ± 0.018	0.001 ± 0.000	0.001 ± 0.000
CD	0.04 ± 0.00	0.77 ± 0.18	0.14 ± 0.02	0.18 ± 0.01	0.02 ± 0.00	0.12 ± 0.04	0.09 ± 0.01	0.63 ± 0.21	0.01 ± 0.00	0.01 ± 0.00
CRR	10.00	90.00	40.00	23.33	6.67	30.00	16.67	63.33	6.67	36.67

Note: PL (mm), PW (mm), PH (mm), PS (mm^2) and PV (mm^3) respectively represented the paddy grain length, paddy grain width, paddy grain height, paddy grain surface area and paddy grain volume. EL (mm), EW (mm), EH (mm), ES (mm^2) and EV (mm^3) respectively represented the embryo length, embryo width, embryo height, embryo surface area and

embryo volume. SCL (mm^2), VCL (mm^3), VTCL (μm), DTCL (μm), UTCL (μm) and LTCL (μm) represented the surface area, volume, ventral thickness, dorsal thickness, upper thickness and lower thickness of composite layer respectively. CV (mm^3), CD (%) and CRR (%) respectively represented the chalkiness volume, chalkiness degree and chalky rice rate.

Table S4 Paddy grain traits in 30 white rice cultivars

Sample	PL	PW	PH	PS	PV	BV	IHV
d23	8.50 ± 2.20	3.02 ± 0.31	1.94 ± 0.14	71.05 ± 1.02	18.22 ± 1.51	16.21 ± 1.24	11.05 ± 1.21
d24	8.90 ± 0.98	2.86 ± 0.19	2.01 ± 0.17	89.09 ± 10.34	23.23 ± 1.85	20.26 ± 1.59	12.80 ± 0.06
d25	7.63 ± 0.45	2.40 ± 0.08	2.05 ± 0.02	61.36 ± 6.95	14.92 ± 0.23	12.41 ± 0.11	16.84 ± 0.07
d26	8.61 ± 1.52	2.60 ± 0.11	2.05 ± 0.47	70.46 ± 5.87	19.99 ± 0.64	17.72 ± 1.46	11.34 ± 1.03
d27	8.36 ± 0.99	2.54 ± 0.10	2.09 ± 0.26	76.93 ± 9.59	19.31 ± 0.76	17.55 ± 0.32	9.10 ± 0.07
d28	8.62 ± 1.01	2.83 ± 0.12	1.97 ± 0.07	75.21 ± 9.13	19.02 ± 1.99	16.07 ± 1.66	11.52 ± 1.04
d29	9.78 ± 0.25	2.36 ± 0.29	1.83 ± 0.06	68.32 ± 4.92	19.15 ± 2.82	16.64 ± 0.77	13.09 ± 0.34
d30	8.26 ± 0.17	2.53 ± 0.10	1.70 ± 0.05	79.45 ± 5.74	12.15 ± 1.04	10.95 ± 0.20	9.90 ± 1.06
d31	8.25 ± 1.28	2.83 ± 0.11	2.20 ± 0.19	80.58 ± 6.38	23.01 ± 0.94	22.24 ± 1.80	3.33 ± 0.02
d32	7.95 ± 0.16	2.49 ± 0.08	2.01 ± 0.36	70.11 ± 4.11	15.89 ± 1.33	13.81 ± 1.02	13.09 ± 0.04
d33	10.34 ± 1.32	2.43 ± 0.05	2.15 ± 0.04	75.21 ± 1.23	18.42 ± 1.62	16.94 ± 0.51	8.05 ± 1.03
d34	7.80 ± 0.15	2.67 ± 0.10	1.86 ± 0.25	64.87 ± 2.58	15.56 ± 0.09	12.39 ± 0.12	20.37 ± 0.10
d35	8.00 ± 0.16	2.30 ± 0.07	1.91 ± 0.16	65.08 ± 2.32	13.93 ± 1.39	11.84 ± 1.03	14.98 ± 1.11
d36	8.36 ± 0.79	2.70 ± 0.21	2.28 ± 0.19	73.03 ± 6.39	19.45 ± 0.77	17.76 ± 0.66	8.69 ± 0.72
d37	8.56 ± 0.20	2.65 ± 0.10	1.94 ± 0.07	68.37 ± 5.02	17.81 ± 1.09	15.40 ± 1.42	13.53 ± 1.23
d38	7.93 ± 0.16	2.89 ± 0.12	1.97 ± 0.17	63.09 ± 8.34	17.23 ± 1.61	14.13 ± 1.39	18.01 ± 1.45
d39	7.79 ± 0.55	2.74 ± 0.31	2.13 ± 0.02	79.55 ± 4.09	21.39 ± 0.89	20.77 ± 0.81	2.91 ± 0.84
d40	8.66 ± 0.21	2.76 ± 0.32	2.08 ± 0.05	75.05 ± 4.21	21.35 ± 0.94	19.45 ± 1.70	8.89 ± 1.12

d41	8.21 ± 0.18	2.84 ± 0.11	2.14 ± 0.03	64.53 ± 6.88	15.95 ± 1.43	13.21 ± 0.21	17.16 ± 1.11
d42	10.55 ± 1.31	2.50 ± 0.20	2.14 ± 0.08	101.35 ± 6.34	24.68 ± 1.02	21.38 ± 0.94	13.38 ± 1.04
d43	9.16 ± 0.23	2.38 ± 0.09	1.80 ± 0.06	65.63 ± 7.24	17.90 ± 1.65	16.51 ± 0.50	7.78 ± 1.22
d44	7.35 ± 0.64	2.74 ± 0.14	2.04 ± 0.01	66.54 ± 4.09	18.03 ± 0.50	15.42 ± 0.43	14.46 ± 0.34
d45	7.97 ± 0.66	2.51 ± 0.09	2.18 ± 0.22	72.69 ± 2.39	19.01 ± 1.74	16.76 ± 1.61	11.84 ± 1.32
d46	8.53 ± 0.20	2.78 ± 0.41	2.38 ± 0.09	82.28 ± 8.44	21.73 ± 0.93	20.62 ± 0.87	5.12 ± 0.21
d47	10.58 ± 0.32	2.32 ± 0.19	2.05 ± 0.07	81.37 ± 3.26	21.67 ± 2.99	17.88 ± 0.74	17.50 ± 1.0
d48	8.17 ± 0.17	2.50 ± 0.05	2.18 ± 0.13	72.27 ± 4.03	18.99 ± 1.61	18.24 ± 0.51	3.93 ± 0.89
d49	8.20 ± 0.17	2.47 ± 0.02	2.24 ± 0.08	67.74 ± 5.05	17.75 ± 1.55	14.81 ± 1.11	16.55 ± 1.34
d50	9.19 ± 1.20	2.89 ± 0.10	2.14 ± 0.22	74.17 ± 5.14	19.37 ± 1.54	16.69 ± 1.21	13.82 ± 2.18
d51	8.43 ± 0.08	2.76 ± 0.29	2.24 ± 0.17	89.32 ± 4.32	16.59 ± 1.82	14.66 ± 1.55	11.65 ± 0.13
d52	7.84 ± 0.15	2.65 ± 0.08	2.66 ± 0.06	72.34 ± 3.32	14.98 ± 0.23	12.99 ± 0.81	13.87 ± 0.37

Note: PL (mm), PW (mm), PH (mm), PS (mm^2), PV (mm^3), BV (mm^3) and IHV (mm^3) represented the paddy grain length, paddy grain width, paddy grain height, paddy grain surface area, paddy grain volume, brown rice volume and internal hollow volume respectively.
